# “ I can’t do it anymore”: a qualitative study on the emergence of crisis in outpatient palliative care—the perspective of family caregivers

**DOI:** 10.1186/s12904-025-01664-y

**Published:** 2025-02-11

**Authors:** Sofia Azhar, Anne Herrmann-Johns, Daniel Wolff, Michael Rechenmacher, Ulrich Kaiser, Maria Wasner

**Affiliations:** 1 Dept. for Epidemiology and Preventive Medicine, Division of Medical Sociology, University of Regensburg, Regensburg, Germany; 2 Catholic University of Applied Sciences, Munich, Germany; 3 Dept. of Medicine III, University Hospital Regensburg, Regensburg, Germany; 4 Bavarian Center for Cancer Research (BZKF), Regensburg, Germany

**Keywords:** Palliative outpatient care, Family caregiver, Crisis, Qualitative research

## Abstract

**Background:**

The outpatient palliative care system is a central component of the palliative care structure in Germany, with family caregivers playing a vital role in ensuring and maintaining its success. However, crisis situations can destabilise and harm outpatient care. Previous studies have analysed hospitalisations and emergency admissions during palliative care. However, little is known about the factors that contribute to the emergence of crises in palliative outpatient care. The aim of this study was to identify factors contributing to the emergence of crisis in palliative outpatient care including the identification of protective strategies.

**Methods:**

A qualitative study using semi-structured interviews with family caregivers recently involved in outpatient palliative care was performed. The analysis was conducted using thematic coding.

**Results:**

A total of 15 family caregivers (13 female and 2 male) were recruited for the study. Crisis situations emerged through a cumulation of factors. Four categories of factors were identified: (1) structural factors, including limited access to health care professionals and a lack of necessary information, (2) illness related factors such as sudden progression in the illness trajectory and symptoms, (3) the intensity of care involvement and (4) emotional and psychological burden of family caregivers. A diverse range of protective strategies were employed by family caregivers, encompassing the involvement of the social network, the pursuit of information and the identification of a sense of purpose.

**Conclusion:**

The findings of this study confirm the importance of providing continuous, competent, and empathetic care to both family caregivers and patients in palliative outpatient care. The study highlights the importance of expanding the palliative care infrastructure, as well as ensuring unrestricted access to palliative care professionals, developing tailored information tools for family caregivers, and reducing bureaucracy. Further studies are needed to identify additional influencing factors and evaluate effective measures.

**Supplementary Information:**

The online version contains supplementary material available at 10.1186/s12904-025-01664-y.

## Background

In the field of palliative ambulatory care, the role of family members as caregivers is of great importance in supporting patients in their final stages of life [1]. In Germany, palliative outpatient care is available in the form of general or specialised care. General outpatient palliative care is provided by general practitioners or specialists and an outpatient care team with basic palliative care training. Specialised outpatient multi-professional palliative care teams have received specialised palliative care training and provide medical and nursing care for patients with particularly complex needs [2]. The applied definition of palliative care in this study corresponds to the definition provided by the WHO 2002 [3]: *Palliative care is an approach that improves the quality of life of patients and their families facing the problems associated with life-threatening illness, through the prevention and relief of suffering by means of early identification and impeccable assessment and treatment of pain and other problems, physical, psychosocial and spiritual*.

Family caregivers represent a vital element of outpatient palliative care, providing support for approximately 80% of those in need of care in Germany. It is notable that a significant proportion of these caregivers are women, with 60 to 80% of them being female [4, 5]. Family caregivers defined by the Family Caregiver Alliance are informal caregivers, which means, that they are unpaid individuals involved in assisting others with activities of daily living or medical tasks. The care recipient may be for instance a spouse, partner, family member but also friend, or neighbour [6]. These caregivers can be exposed to different burden of care and eventually might be overwhelmed, which can lead to crisis situations in the palliative outpatient setting [7–11]. Existing studies indicate that considering caregivers needs is crucial for understanding the emergence of crisis in the palliative outpatient care setting [12].

The transactional stress model of Lazarus and Folkman provides a framework for understanding the emergence of stressful situations. According to this model, individuals subjectively assess their situation, which includes the resources available to cope with it. Based on this assessment, they may experience the situation as manageable or overwhelming [13]. Research indicates that family caregivers can develop a range of coping strategies to manage multiple stressors [14–19]. However, caregiving can also be perceived as a burden, leading to anxiety, depression, physical symptoms (such as back pain or sleep disturbances), and social problems (such as loss of income and social isolation) [20].

While previous psychological research has emphasised problem-focused coping strategies, recent studies suggest that a combination of problem-focused, emotion-focused, and cognitive strategies may be more effective to cope with stressors, rather than relying on a single coping strategy [21].

Organisational research from various disciplines was among the first to analyse crisis situations to understand their causes, preventive measures, and evaluation of outcomes. The results of these analyses indicate that crisis situations are part of larger processes rather than discrete events. This finding is consistent with psychological stress models such as the cumulative stress model [22]. Additionally, the research has identified involving stakeholders in identifying risk factors as a measure to prevent crises in organizations [23–25].

The analysis of crisis situations in the context of palliative outpatient care can facilitate an understanding of the underlying causes and mechanisms that precipitate such crises. Furthermore, it can assist in the identification of potential clinical implications and the development of strategies for the prevention of such occurrences. When analysing crisis situations in the palliative outpatient care setting previous studies focused on the quantitative analysis of hospitalisations and emergency admissions in the palliative outpatient setting [26–31]. This study analyses the micro level of crisis situations in palliative outpatient care from the perspective of family caregivers. This micro level analysis allows a more precise and deeper understanding of the emergence of crisis situations. The aim of this study was to identify factors contributing to the emergence of crisis in the palliative outpatient care including the identification of protective strategies.

## Methods

### Study design

The study employed a qualitative exploratory approach using episodic interviews with family caregivers in the palliative outpatient setting [32]. This exploratory design was selected due to the paucity of existing knowledge on this topic and the desire to gain insight into the lived experiences of family caregivers in crisis situations.

### Setting

The study was conducted in Bavaria, the largest German federal state (13 369 393 inhabitants). Palliative outpatient care in Bavaria is comprised of non-specialised palliative care (AAPV), which encompasses nurse-led outpatient care, physicians, other providers such as ambulant hospices and therapists who have undergone basic training in palliative care. It is intended that this care will meet the needs of 90% of patients requiring palliative care. The remaining 10% receive care from specialised teams (SAPV) with advanced palliative care knowledge. These teams are responsible for patients with complex symptoms who require advanced care. In cases where patient's palliative care needs are beyond the scope of outpatient services, they can be admitted to a palliative care ward of a hospital [2]. The structure of palliative outpatient care in Bavaria is characterized by a low density of outpatient physicians with training in palliative medicine (3,8 per 100,000 inhabitants), a low number of specialised outpatient care teams (3,6 per 1,000,000 inhabitants) and a low number of ambulant hospices (1,1 per 1,000,000 inhabitants) [2, 33, 34]. In the context of outpatient palliative care in Bavaria, family caregivers are entitled to receive governance support in the form of family care time for a maximum duration of 24 months. During this period, they are not remunerated by their employer but are financially supported through a state loan, which is to be repaid. Additionally, family caregivers are eligible for a three-month leave from work for the purpose of providing care in the final phase of life [35].

### Sampling

Recruitment started in 09/2021 and ended in 09/2022. 36 palliative care facilitators (specialised outpatient palliative care providers, outpatient nursing services, hospice associations, self-help groups and associations involved in palliative care) in Bavaria were selected from an official website of the DGP (Deutsche Gesellschaft für Palliativmedizin), the German palliative care association [36].

These 36 facilitators were subsequently contacted via mail and after a two-week interval, via telephone. 23 service providers consented to participate in the study. They were provided with informational materials for dissemination among potential participants. In addition, service providers were offered an on-site presentation about the study. The facilitators established contact between the researcher and the family caregivers. Fifteen family caregivers from 9 service providers agreed to participate in the study. There was no information given by facilitators whether family caregivers refused to participate.

The study included adult caregivers (≥ 18 years) in a palliative outpatient care setting who met the definition of family caregiver as outlined by the Family Caregiver Alliance and who had experienced a crisis during the previous two years [6]. All participants met the inclusion criteria, no one was excluded.

The study employed a purposive sampling strategy. The factors identified from the literature as influencing the experience of palliative outpatient care were taken into account during the sampling process. These included the type of care provided (specialised or non-specialised), the location of the care (urban or rural), the relationship between the patient and the carer, the nature of the patient's illness, the ethnicity and gender of the patient and carer [31, 37, 38].

### Data collection

Episodic interviews were conducted combining the request of episodic and sematic knowledge including definitions and narrative prompts [39]. The combination of semantic and narrative segments allows a more natural conversation flow and a triangulation of different approaches to collect different sorts of data [40]. Episodic interviews allow to access both forms of knowledge about a subject by collecting both narrative-episodic knowledge through narratives and semantic knowledge made accessible by correct pointed questions. This combination leads to a triangulation of approaches including different types of data: argumentations, subjective definitions, examples, narratives of situations, stereotypes, repisodes. Semantic knowledge is presented in an argumentative and theoretical presentation, whereas episodic knowledge is presented in a narrative format [32]. The definitions of crisis situations in palliative outpatient care in the present interviews are derived from the individual experiences of the participants, as narrated in the episodes.

A semi-structured interview guide (Table 1) was developed as a data collection tool based on the episodic interview approach and current literature on palliative outpatient care [31, 41–43]. The interview guide was piloted by SA before the start of the study (Table 1) with the pilot case being excluded from the final data set. The piloting led to the change of the first interview question and the wording. Data collection continued until no new themes were evident in three consecutive interviews and thus theoretical saturation was reached.

**Table 1:** Interview guide summary

• Can you tell me how the palliative care is/was organized? • Who is/was involved? • How has the palliative outpatient care been initiated? • Please tell me what is a crisis in the palliative outpatient care from your perspective • How did this situation occur? • Please tell me what happened in this situation • Did you have a document for emergencies /Advance care plan/health care plan? • How did you react? • What might have helped in this situation? • Which barriers have you been confronted with? • Which experiences did you have before this situation with death and dying? • How did this experience change your behaviour/perspective ? • Which consequences do you take from this experience?	

Additionally, participants completed a questionnaire providing sociodemographic information to describe the sample. The interviewer SA, recorded field notes before and after each interview, documenting expectations, specifics, concerns, and the researchers' reflections. These filed notes were used for the dense description in the individual case studies and comparative analysis of the interviews. The interviewer has worked in the palliative care field and is experienced in qualitative research in this field. Given the interviewer’s established rapport with family caregivers, a conducive environment for data collection could be readily established.

### Analysis

Interviews were tape recorded with participants consent and transcribed verbatim in German language by SA using the transcription system of Fuß and Karbach [44]. This transcription system allows the transcriptionist a flexible adaptation of transcription rules depending on the focus of the analysis. In this study the interviews were transcribed verbatim with a light language smoothing and the inclusion of pause, speech, vocalisations, non-speech vocalisations, interactions and interruptions. The punctuation is in simplified intonation. The initial author translated citations back and forth in English using the deepL translation service, and these translations were subsequently confirmed by the co-authors. The interviews were analysed by the Thematic Coding approach by Flick containing the previous described steps: initiating text work, thematic structuring, thematic and selective coding, comparison of contrast groups and creation of a theory [39]. The procedure of thematic coding is shown in Table 2. Thematic coding is a method to compare different groups regarding an experience and therefore seems to be appropriate method to analyse the episodic interviews. These interviews were coded by SA in the data analysis programme MaxQDA2022 after pseudonymisation.The development of the codes was the subject of deliberations with the co-authors, M.W., A.H. and D.W., as well as with researchers with extensive experience in the field of palliative care. Subsequent to these discussions, the codes were revised and augmented. In order to ensure trustworthiness, credibility, transparency, transferability and rigour in the present study the *Four-Dimensions Criteria* (FDC) of Lincoln and Guba have been applied [45].

**Table 2:** Thematic coding procedure [39]

1. Short description of each case	Includes: Statement of the interview, information about the person, central topics mentioned by the interviewee. These case descriptions form part of the results
2. Developing a thematic structure	Case analysis of every case to develop system of categories (open and selective coding). Selective coding is used to generate thematic domains and categories. The cross-checking of categories and thematic domains results in a thematic structure
3. Fine interpretation of thematic domains	Single passages of texts are analysed in greater detail using key questions (conditions, interactions, strategies, consequences). Similar codes in individual groups and specific topics are elaborated. Generalisation is based on the comparison of cases and groups and aims at the development of theories

### Ethics

The study was approved by the Ethics Committee at the University of Regensburg, Germany (21–2506-101). All participants signed an informed consent to participate. Participants were made aware of the ability to ask questions and terminate the interview at any time. Furthermore, the participants were apprised about the strict confidentiality of the interview. Moreover, the family caregivers were provided with a contact address for psychosocial support.

## Results

A total of 15 interviews were conducted, 13 of which were done by a phone call and 2 were conducted at the participants' homes. The location of the interviews was at the discretion of the participants. Due to the circumstances of the study, which was conducted during the pandemic of the Coronavirus, the participants were given the option of conducting the interviews via telephone. The utilisation of telephone interviews enabled participants to maintain a greater distance from the interviewer, thereby facilitating more open and candid responses. Furthermore, the participants were able to conduct the interviews in a location that they found conducive to their comfort and wellbeing, such as while engaged in a leisurely stroll.

The interviews lasted a mean of 32 min (range: 17–60 min). Four follow-up interviews were conducted to address additional questions that arose during the analysis.

### Sociodemographic data

The study participants were predominantly female (*n* = 13). The two male participants provided their perspective as the son of a patient who did not reside in the same household. The female participants provided their perspective as the mother (*n* = 2), wife (*n* = 6), parent (*n* = 2), child (*n* = 2) and daughter-in-law (*n* = 1) of the patient. Tables 3 and 4 provide details on the characteristics of both the caregivers and the patients.

**Table 3:** Sociodemographic characteristic of the participants *n* = 15

Age mean (range)	59,3 years (37–75)
**Sex (n)**	
Female	13
Male	2
**Highest school-leaving qualification (n)**	
Secondary school	8
General school	3
Higher school certificate	4
**Living situation (n)**	
Lived in the same residence as the patient	8
Lived in another residence as the patient	7
**Relationship to the patient (n)**	
Spouse	6
Child	6
Parent	2
Daughter-in-law	1
**Experience in care (n)**	5

**Table 4:** Sociodemographic and disease related characteristic of the patients

Age mean (range)	70 years (29–88)
**Sex (n)**	
Female	5
Male	10
**Disease (n)**	
Malignant d	11
Multimorbidity	1
Neurodegenerative d	3
**Symptoms**	Dyspnoea, pain/ lying pain, aggressive behaviour, restlessness, state of panic, diarrhoea, disturbance of coordination, aphasia, bleeding, increased salivary secretion, immobility
**Type of care (n)**	
Specialized palliative care	9
General palliative care	6
**Hospital admissions (n)**	5 (4 male patients)

The following Table 5 provides an overview of the themes which emerged in the interviews.

**Table 5:** Overview of themes

Emergence of crisis	The crisis situation-factors leading to crisis				Strategies to overcome crisis and wishes
• Context of crisis • Acute and cumulative crisis	Structural factors • Distance to providers • Access and communication with health care professionals • Rotating staff • Bureaucracy	Illness related factors • Symptoms • Illness trajectory • Disease-dependent access to specialized palliative care	Emotional and psychological burden of family caregivers • *Emotional burden* • *Psychological burden*	Intensity of care involvement • *Living in the same household* • *Sleep deprivation*	• *Involvement of social network* • *Searching for information* • *Learning new skills* • *Regeneration* • *Finding a meaning and further psychosocial support* • *Hospitalisation*

### Emergence of crisis

As the phenomenon of crisis is a subjective perception of individuals, constructed by their individual circumstances and experiences, participants were first asked to describe what crisis in palliative outpatient care meant to them. Most participants described crisis situations as a state of being overwhelmed, using phrases such as *“All you can do is stand there and do nothing*” (T13, l.43), “One *centimetre from collapsing” (T3, l.128),* and *“a big disaster” (T10, l. 172).*

The crises described by the interviewees did not arise from a single cause, but rather from a combination of various factors that came together during the crisis.

“[…] The point was insidious. I noticed because I couldn't sleep at night […] and I even became impatient the next day. And that's just not possible with a sick person.” (T19, l. 203–206).

Crisis situations were triggered by acute incidents that required immediate action. Caregivers stressed the importance to be prepared for these situations in advance to be able to get appropriate help and guidance. Family caregivers felt particularly anxious and unsure of appropriate reactions in these situations, when faced with life threatening symptoms such as heavy bleeding or rapid deterioration of the patient's condition. Additionally, crisis situations were triggered by the constant presence of burdensome factors during a period when the patient required intensive support.

A mother of a patient with amyotrophic lateral sclerosis:

“So, a crisis, let's put it this way, it was for me now when my daughter had shortness of breath and I didn't know how to deal with it. That was a crisis for me.” (T9,l. 29 f.)

**Fig. 1:**
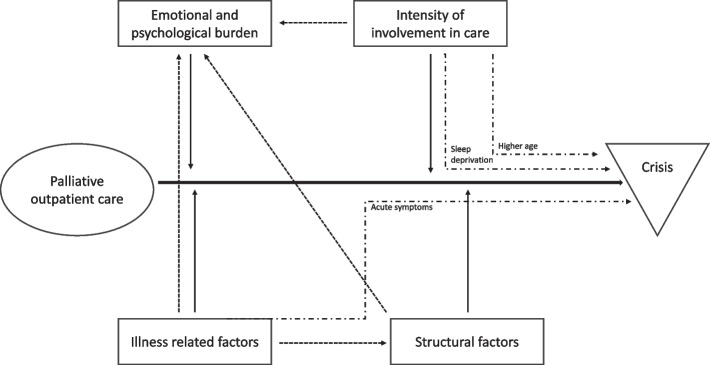
Process of crisis emergence in the palliative care setting  symbolize catalysts in the emergence of a crisis

The analysis of the reported crisis situations revealed four main factor categories that influenced the emergence of crisis situations: structural factors, illness-related factors, the intensity of care involvement and the emotional and psychological burden of family caregivers. These four factor categories appeared to interact and mutually reinforced each other. Additionally, acute symptoms in patients and sleep deprivation and higher age in family caregivers acted as catalysts in the emergence of a crisis (Fig. 1).

### Factors influencing the emergence of crises

#### Structural factors

The most identified factors pertained to the organisation and structure of the palliative outpatient care.

#### Distance to providers

In Germany, not all outpatient care teams are permitted to provide specialised care such as intravenous infusions and tracheostoma care due to the lack of training. Moreover, interviewees in rural areas reported a too large distance to specialised healthcare providers, which prevented homecare by specialist palliative care teams. Furthermore, the distance prevented palliative care teams to perform daily visits or permitted only once daily visits in patients with high support needs.

"Because as I said. They tried to find a care service, but for this issue [tracheostoma] there is no one in the region who does it. The nearest one is then about thirty to forty kilometres away.” (T19, l. 61–63).

#### Access and communication with health care professionals

In some crises, family caregivers lacked the necessary knowledge and information to cope with overwhelming situations. In such circumstances, having a designated contact person with expertise in palliative care would have provided a sense of security. Furthermore, it would have been beneficial for the care team providing palliative care to offer information regarding the illness trajectory, the dying process, drug administration, and alternative care structures.

Access to healthcare professionals was primarily dependent on the availability of a specialised palliative care service (SAPV = Spezialisierte Ambulante Palliativ Versorgung) providing outpatient care. These specialised teams offer a 24-h on-call and operational readiness, which provided family caregivers with a sense of security.

Family caregivers who received regular updates on their loved one's condition and progress, as well as information about advance care documents and individual needs in the form of regular calls and personal conversations with the care team, rated this experience positively.

“So, I was virtually always supplied with information …. I found that very, very good, because there was just trust there.” (T10, l. 264f.)

In contrast, family caregivers found it challenging to access healthcare professionals from non-specialised palliative care teams which burdened them. Moreover, a lack of palliative care competence, including proficiency in drug administration and communication with patients and family caregivers, was identified as a contributing factor to the occurrence of stressful circumstances.

#### Rotating staff

The interviewees identified a lack of continuity of care-providing staff as a burdening structural factor that impeded the implementation of a consistent care plan. As a result, the lack of familiarity of the facilitators with the patient resulted in a dearth of crucial information regarding the decision-making and action required in crisis situations. In instances necessitating immediate action, the inexperience of caregivers in dealing with patients could precipitate crises and even inflict harm upon the patient. This resulted in family caregivers being unable to rely on the support of professional caregivers.

“We (the patient's mother and the nurse) had the feeling that we had to open her jaw a little because it was grinding terribly. The nurse was a bit…yes, overwhelmed. […]. And that was […] not such a good idea because we almost hurt her a bit.” (T9 l.59–67).

#### Bureaucracy

Interviewees complained about hurdles of bureaucracy to get the equipment for palliative home care including long processing times. The care provision without equipment led to a physical burden in family caregivers or made it even impossible to provide the task. A participant narrated that she applied for equipment and was retraumatized when the application was granted after her husband died.

“I was unable to get the wheelchair onto the terrace, and the health insurance company was very slow. The palliative care provider promptly wrote me a prescription for a ramp. After the death, I was informed that the medical service would assess whether the ramp was necessary. […] This still upsets me.” (T18 l. 113–122).

### Illness related factors

The character and symptoms of the illness itself influenced the emergence of crisis situations described in the interviews.

#### Symptoms

Diseases that cause personality changes and restlessness were perceived as burden for family caregivers especially during advanced disease phases, when the patient's health declined, and symptoms increased. Coping with the personality changes of the relative and the burden of a 24-h presence due to the patient's high support needs caused psychological distress.

A participant phrased this aspect in this way:

"[…] but what one reflects somehow only later this blatant change of personality […] So and that was actually for us I would say the most difficult […]"(T15, l. 46 f.).

The occurrence of acute symptoms, such as dyspnoea or bleeding, triggered crisis situations that required immediate action from family caregivers and led to uncertainty and anxiety in caregivers. Most patients with acute symptoms were cared for by specialised palliative care teams and were supplied with emergency medicine.

#### Illness trajectory

The lack of predictability, especially in illnesses with an ongoing gradual decline such as organ failure and a neurocognitive decline due to dementia, posed a challenge for family caregivers who were not familiar with the healthcare system. This necessitated a high degree of flexibility and a comprehensive understanding of the array of available services from the family caregivers.

“[…] It is incredibly difficult for a relative to get help in time […] The institutions exist, but they don't come to you because they now realize that there is a crisis […].” (T10, l. 141–143)

#### Disease-dependent access to specialised palliative care

The patient's illness affected their access to specialised palliative care, which is apparent in the cases included in the analysis. Patients with cancer or motor neuron diseases received support from a specialised palliative team comprising healthcare professionals with expertise in this area (SAPV). These teams provided coordinated care and 24-h accessibility. However, patients included in the interviews with non-malignant primary diseases were usually cared for by non-specialised palliative care teams. The participants described cases in which patients with non-malignant diseases exhibited severe symptoms such as dyspnoea and pain that would have been mitigated with specialized care.

### Emotional and psychological burden of family caregivers

Most interviewees reported about the emotional and psychological burden of their caregiver experience which influenced the emergence of crisis.

#### Emotional burden

The emotional attachment to the patient caused a burden for family caregivers and affected the provision of care for the patient. Interviewees who had cared for others before reported that caring for a close family member was a more challenging task.

“[I have] accompanied my grandmother […], my father […], and [patients] in my profession. However, it was difficult for me … because my mother suffered […]. “ (T5 l.253–255)

The interviewed wives reported experiencing anticipatory grief and emotional closeness to their partner, which made caregiving burdensome. These wives had been in a long marital relationship with the patient and wanted to reciprocate and express gratitude for the fulfilling relationship, as well as fulfil their partner's wish to die at home. As a result, they sacrificed their own health to fulfil their partner's wish resulting in role conflicts, psychological and physical burden.

The same conflict was reported by a mother reporting her feelings during her child's dyspnoea:

“[…] you could see [the shortness of breath] and of course that was always a feeling where I … yes (cries) sometimes I would have liked to run away. “(T9 l. 110f.)

Of note, male caregivers did not directly express their psychological distress. However, they did express their distress indirectly, for example when they told, that it was hard for them to watch the decline of their relatives they cared for.

Family caregivers also experienced emotional burden when the patient did not accept their care due to personality changes or familial conflicts. In such cases, caregivers had to provide care despite the patient's resistance, which led to feelings of undervaluation and caused significant emotional distress.

“She (the patient) only argued […]. It wasn't easy at the beginning. She was…protesting.“ (T7 l. 76f.)

“[…] that was actually the most difficult thing for us, I would say, that you can no longer […] explain certain things to the patient who has changed due to illness.“ (T15 l. 49f.)

#### Psychological burden

The interviewees identified several behaviours of healthcare professionals that contributed to a lack of trust in facilitators. These included situations where professionals were uninformed about the patient's care plan or diagnosis, lacked knowledge of how to correctly apply medicine in palliative care, and showed a lack of empathy towards family caregivers causing significant psychological burden.

An interviewee recounted an incident in which a physician accused the family caregiver of calling emergency services when the patient was experiencing heavy bleeding at night. The physician's response demonstrated a lack of empathy for the family caregiver in this situation:

"There I would like to see you at one o'clock in the night, when the blood comes out of the cannula here, whether you then do not call the emergency doctor, if you are all alone with your partner in the house […] So then I should have let him bleed to death according to [the doctors] opinion."(T19, lines 155–159).

### Intensity of care involvement

The intensity of care involvement did influence the emergence of crisis situations described by family caregivers.

#### Living in the same household

Interviewees who lived with the patient and provided a high level of support in daily activities reported a significant physical burden of care due to a 24-h presence. The spouses of patients were responsible for caring for their husbands for an extended period. During interviews, these individuals reported experiencing a range of symptoms, including gastrointestinal complaints and joint pains, which impeded their capacity to provide care. This, in turn, destabilized the outpatient care system, as the maintenance of palliative outpatient care depended on them. The interviewees also mentioned their age as a contributing factor to physical limitations. Children of patients who lived in the same household intermittently also reported physical burdens, but not long-lasting physical or psychosomatic symptoms.

An interviewee describes how she mobilized her husband and became physically strained:

“So I did this [mobilization] very, very often for months and the doctors say that's probably why I started to get knee pain and I started to have massive knee pain from [time point] on […] I suffered terribly.” (T16, l. 119–122).

Interviewees who were not living in the same household did not describe the physical burden of care. These interviewees were more involved in the organisation of care and communication with health care providers and described crisis from an organisational perspective. The intensity of caregiver involvement did not depend on the type of outpatient palliative care, since specialised palliative care teams did not provide physical care.

#### Sleep deprivation

Continued care during nighttime leading to sleep deprivation was identified by all caregivers who lived in the same household with the patient as a contributing factor to crisis situations. Sleep deprivation affected their performance during the day, reduced caregiving capacity and resulted in impatience with the patient. These effects catalysed the emergence of crisis situations since they reduced the resilience of family caregivers.

“The challenge is the nights […] that was the challenge. There is simply no rest.” (T18 l 100f.)

### Strategies to overcome crisis and wishes

#### Involvement of social network

Caregivers who had a broad social network and were able to distribute workload related to the care they provided to the patient reported greater ability to regenerate. Nevertheless, the majority of the interviewed spouses reported being mostly isolated from their social network during the illness of their husbands. They expressed a desire for more social support, such as the presence of an additional care provider who could accompany the patient or a neutral individual with whom they could discuss their concerns when their husbands were unable to engage in conversation due to illness.

In contrast, children of patients maintained their social network when caring for the patient and were able to distribute care tasks. The involvement in social networks including multiple family members or professional specialised aid organisations seemed to be an effective coping strategy.

A participant reported about the organisation of care in her family:

“[…] on the one hand, we alternated in terms of time, but on the other hand, we also alternated with the activity. So, when it came to turning {the patient}, my husband did that because he clearly has more strength.” ( T13, l. 100f.)

#### Searching for information

If family caregivers did not have a designated contact person for issues related to patient care, they attempted to obtain information through various means, such as reaching out to friends who work in the medical field, searching for relevant organizations, or conducting online research. An interviewee reported about her strategies:

"[…] where we were always completely at a loss as to what to do and then I kept googling for days on end and talking to a friend who works in the hospital and you kind of tried to help yourself because you don't have a contact person" (T3, l. 55f.)

The respondents expressed a desire for a professional, specialized contact person who could provide them with the information they require and assist them in decision-making for the patient. They also expressed their desire for a contact person they are familiar with and who knows the family situation and the caregiver well. This would enable them to establish a trusting relationship with the patient and the family caregiver. Furthermore, they indicated a need for a care coordinator who would oversee the care situation and coordinate the care at home.

#### Learning new skills

To maintain palliative outpatient care, family caregivers often learned caregiving skills from professional caregivers or friends. These skills included mobilization, drug administration, tracheostomy care and wound care. However, these learning experiences were often involuntary and resulted in physical and psychological distress for family caregivers. The involuntary learning experience was a consequence of the lack of availability of specialized care teams, particularly in rural areas, and when patients had non-malignant diseases.

#### Regeneration

Having the ability to leave the caring situation, to sort thoughts and regenerate was described by all participants as an important coping tool.

A caregiver described the regenerative effect of her gardening:

“You practically notice [the physical degeneration] when you're always together for twenty-four hours straight, as I said. You can directly see how the whole thing… goes downwards. Fortunately, I have our garden. I can keep myself busy there too. That's my recovery.” (T17, l. 238–24).

The level of regeneration varied among the interviewees. Children of patients were able to return to their own homes and communicate with friends and family, while wives of patients often opted for regeneration methods close to the patients, such as gardening, taking short walks with their dogs, or reading books.

#### Finding a meaning and further psychosocial support

Family caregivers have sought to mitigate the distressing nature of their role by, for instance, establishing organisations to assist others in similar circumstances. They have also reflected on their own values and ideas, expressing gratitude for the opportunity to spend time with the patient and fulfil their wish to die at home.

An interview participant described her gratitude for fulfilling her husband’s wish:

“I am so happy that I was able to fulfil his wish to die at home. That's what he always wanted and that's what he always said. Don't put me in hospital, don't.” (T.20, l. 312ff.)

Female family caregivers indicated a desire to utilise professional psychological support to cope with the psychological and emotional burden. However, the distances involved in accessing such support, coupled with lengthy waiting periods for appointments, impeded their ability to do so.

“What I would have liked […] was more psychological support […] I went to a chaplain in a parish, but I didn't get a psychologist. So, I called around and there just isn't one. […] That's what I would have liked to see in our mental health services.” (T13, l. 148–156).

#### Hospitalisation

If the physical and psychosocial burden of care overstressed family caregivers, inpatient services like hospices or palliative care units were utilized. The transfer to these inpatient services was organized by specialized outpatient care services or the attending family doctor.

A family caregiver caring several years for the patient:

“[…] I turned to the SAPV and said I can't go on any longer. I can't do it anymore. That was the first time in all these […] years. Where I admitted to myself. I can no longer manage it on my own.” (T19 l. 194–196).

## Discussion

The study aimed to develop a framework model to understand the emergence of crisis situations in outpatient palliative care. Several factors were identified that, in combination, contributed to crisis, including structural factors, illness-related factors, the intensity of care involvement and the emotional and psychological burden experienced by family caregivers.

Participants identified several structural factors contributing to crises in palliative care. The health care system including palliative care in Bavaria is characterised by significant distances between healthcare providers in rural areas and a low density of specialised outpatient care teams, specialised palliative care teams and physicians with training in palliative medicine [46]. In accordance with the framework agreement for the provision of specialised outpatient palliative care, insured individuals who require specialised palliative care are characterised by the presence of complex symptoms and an incurable, progressive disease that has advanced to the extent that life expectancy is limited. The legislator currently assumes that approximately 10% of individuals who are dying require the services of an specialised palliative care team [33, 47].

This absence of available healthcare providers placed a burden on family caregivers, since it prevented patients from receiving treatment from the specialised care teams at home who in consequence had to rely on local non-specialised resources. The development of regional networks to provide palliative care in rural areas is therefore important to support family caregivers and prevent the emergence of crisis situations [48]. Digital health devices can potentially aid to optimise the utilisation of human resources and communication with staff, patients and caregivers in the outpatient palliative care setting [49]. Furthermore, the study revealed that family caregivers frequently requested access to designated healthcare professionals in order to obtain information that would enable them to cope with the situation. This included information about the illness trajectory, the dying process and information about services. These findings are in line with a previous study which demonstrated that caregivers who had continuous access to a contact person who was aware of their situation reported a more positive care experience [50].

In cases where interviewees did not have access to designated contact persons, the use of alternative information resources, such as the internet may be of help. Existing literature indicates that although internet-based information for caregivers is abundant, identifying necessary information provided with a individually tailored content can be challenging, and the content may be either too general or too detailed [51]. The development of open access online resources, in consultation with family caregivers, such as CareHelp, could help better provide tailored information to meet their needs [51]. Another effective measure may be the systematic evaluation of the needs of family caregivers using standardized scales, such as the Carer Support Needs Assessment Tool, by palliative outpatient care providers. This systematic assessment can help to provide individual care and promote the development of a trusting relationship with the family caregiver [52].

Another structural factor that emerged during the interviews was the bureaucracy within the palliative outpatient care system. The family caregivers who were interviewed reported bureaucratic application processes for medical equipment and outpatient services related to palliative care. Barlund et al. reported similar findings regarding the bureaucratic challenges faced by family caregivers when applying for attendance allowance at the Norwegian Labour and Welfare Administration [53]. The reduction of bureaucracy and shorter responding times could assist family caregivers in providing and maintaining outpatient care, thereby reducing healthcare costs [54].

The character and symptoms of an illness itself influenced the emergence of crisis situations described in the interviews.

Acute symptoms such as dyspnoea or bleeding acted as a catalyst, often triggering acute crisis situations including unscheduled hospital admissions. This finding is consistent with an analysis of hospital admissions, which identified uncontrolled symptoms as a main reason for hospitalisation in cancer patients [27]. Two approaches could be employed to improve management of acute symptoms in palliative outpatient care. Numico et al. propose that the healthcare system should prioritise the development of efficient services for patients with palliative care needs in hospitals [27]. An alternative approach may be the utilization of early warning systems in the outpatient palliative care setting, which monitor the evolution of symptoms in patients to permit early outpatient interventions [54].

In this context it should be considered, that in Germany the patient's illness affects the access to specialised palliative care with patients suffering from non-malignant diseases or diseases with complex illness trajectories, such as hematologic malignancies, facing greater difficulties accessing specialised palliative care despite similar burden compared to cancer patients [31, 38, 48]. Of note, the majority of interviewees in our sample were family caregivers of patients with malignant diseases.

Another distressing symptom identified by carers was personality changes and agitation in patients at advanced stages of the disease. These symptoms led to psychological distress for carers as they observed changes in close family members in anticipatory grief and had to be constantly present for the patient with high support needs. It is widely acknowledged that family caregivers of persons with dementia experience high levels of burden [55]. Studies of male carers of people with dementia show that those who experience positive aspects of caring feel affirmed in their commitment to their marital relationship [55]. Marital bonds that are strong and positive can support good adjustment in caregivers [21]. A study demonstrated that spousal caregivers who perceived greater appreciation from their partners for their assistance exhibited enhanced psychological well-being. Consequently, couple-focused approaches may prove an efficacious intervention in reducing psychological distress in family carers and in reducing the occurrence of crises in outpatient palliative care [55, 56].

The emotional attachment to the patient caused a burden for family caregivers and affected the provision of care for the patient. In this study, family caregivers experienced emotional distress due to their proximity, which interacted with other factors causing crisis situations in the palliative care setting [56–59].

Another source of psychological distress for caregivers was their inability to delegate their perceived responsibility for the patients to the facilitators. In this context participants of the study reported that trust between themselves and healthcare professionals was impeded by frequent changes of staff, instances of unprofessional or incompetent care, and a lack of empathy towards caregivers. An analysis of the association between caregiver worries and psychosocial well-being revealed a significant positive correlation between worry and physical symptoms, as well as depressive and anxious symptoms, and strain levels in family caregivers of home-cared cancer patients [60]. Existing studies confirm the importance for healthcare professionals to provide continuous, competent and empathetic care to build trust with family caregivers [48, 53, 61]. This can be enhanced by thorough communication training and training in professional palliative care.

Only female caregivers in the study reported a desire to use professional psychological support to cope with their psychological burden. This could be attributed to male caregivers' reluctance to express their emotional distress which has been previously described in the literature or to the fact that female caregivers experience a greater burden than male caregivers [55, 58, 59]. Previous research has demonstrated that even brief educational interventions can alleviate distress among caregivers in the home-based palliative care setting [62].

In this study, interviewed wives reported a lack of support of their social network and negative impacts on their health due to caregiving. These wives had already been caring for the patient at home, isolated from social support for a long time before the crisis occurred and reported physical and psychosomatic symptoms. The absence of social support for female caregivers may stem from societal expectations on women, leading family members to perceive less need for support and therefore less likely to offer it [58, 63]. An effective intervention could involve assisting caregivers in accessing support from their social network, such as assertiveness training and communication skills [64].

All family caregivers who resided in the same household as the patient during the crisis reported physical burden. This is consistent with existing studies that examined the effects of co-residence on caregiver outcomes [65–67]. A vulnerable population in particular are older wives with displace and higher response to stressors leading to prolonged physical and psychosomatic distress [21]. An additional stressor was the need of a 24-h care in care providers living with the patient resulting in sleep deprivation.

Affected care providers reported a decline in their performance during the day, reduced caregiving capacity, and impatience with the patient due to sleep deprivation which is in line with a previous publication [68]. Innovative approaches, such as the development of overnight palliative care services in addition to day hospices, could help to support carers at night and maintain carers' health [69, 70].

## Limitations of the study

For this study the *Four-Dimensions Criteria* (FDC) of Lincoln and Guba were applied, consisting of credibility, dependability, confirmability and transferability and is respectively equivalent to quantitative criteria of internal validity, reliability, objectivity and external validity or generalizability [45].

One method of achieving credibility is through the application of triangulation. The present study employed a single method, which entailed the collection of data of diverse types using the triangulation method. Furthermore, credibility in this study has been enhanced through the process of peer debriefing.

In terms of dependability, it is recommended that a comprehensive description is provided. In this study, the interviewer took field notes and provided detailed descriptions of the cases for inclusion in the case descriptions.

In the present study, the concept of confirmability was ensured through the implementation of a reflexive process by the researcher.

The participants were given the option of conducting the interviews via telephone, given the circumstances of the study, which was conducted during the ongoing COVID pandemic. It should be noted that this method of data collection was not without its disadvantages. The utilisation of telephone interviews imposed constraints on the conveyance of emotional facial expressions. A further limitation of the telephone interviews was the lack of visual access to the environmental circumstances of the interviewees. This necessitated the incorporation of supplementary inquiries into the interviewees' environment and the conditions of their care.

This study used a purposive sampling strategy with a heterogeneous sample derived from the literature [31, 37, 38]. The study also aimed to include a diverse ethnic sample with an ethnicity and cultural background other than German, which could not be achieved. This may be explained by the low utilization of hospice and palliative care services within this group [71].

In order to facilitate the transferability of the results of this qualitative inquiry to other contexts or settings, efforts were made to achieve saturation. Saturation was achieved through the constant review of field findings and the analysis of these findings with an emphasis on the themes in response to the research questions for the study, which involved 15 interviews. As no new themes emerged in three consecutive interviews, it was deemed that the sample size was sufficient.

## Conclusions and implications

The study emphasises the necessity for family caregivers to have enhanced access to structured palliative professional care. This underscores the need to expand palliative care infrastructure, including increased access to palliative care professionals, tailored information tools for family caregivers, the minimisation of bureaucratic procedures and the creation of innovative healthcare structures that are aligned with the needs of family caregivers and demographic shifts. Examples of such structures include overnight palliative care services and community care concepts. With regard to the management of acute symptoms, the application of technical devices to track and evaluate symptoms, and the development of efficient palliative care services in hospitals for patients with acute symptoms, may be effective tools. In order to implement effective preventive measures and coping strategies, it is essential that palliative care facilitators undertake a systematic evaluation of personal crisis factors. These factors include the emotional and psychological burden experienced by family caregivers, as well as their regenerative capacity. Furthermore, it is imperative to implement interventions that facilitate access to support for female caregivers. With respect to the psychological burden, a systematic assessment of caregiver burden through the utilisation of standardised assessment tools is essential. The provision of competent and empathetic care to both family caregivers and patients is of paramount importance. Additionally, it is crucial to ensure access to psychological support for family caregivers.

Further research is required to identify additional influencing factors, such as the structure of health care systems, ethnicity, cultural values and income, and their interactions, and to evaluate effective measures using interventional research.

## Supplementary Information

Additional file 1. Interview guide.

## Data Availability

No datasets were generated or analysed during the current study.

## Abbreviations

DGP: Deutsche Gesellschaft für Palliativmedizin
SAPV: Spezialisierte Ambulante Palliativ-Versorgung
