# Supplementary figures and images for: “ I can’t do it anymore”: a qualitative study on the emergence of crisis in outpatient palliative care—the perspective of family caregivers
Source: BMC Palliat Care. 2025 Feb 11;24:39. doi: 10.1186/s12904-025-01664-y (PMC11818284; doi:10.1186/s12904-025-01664-y)

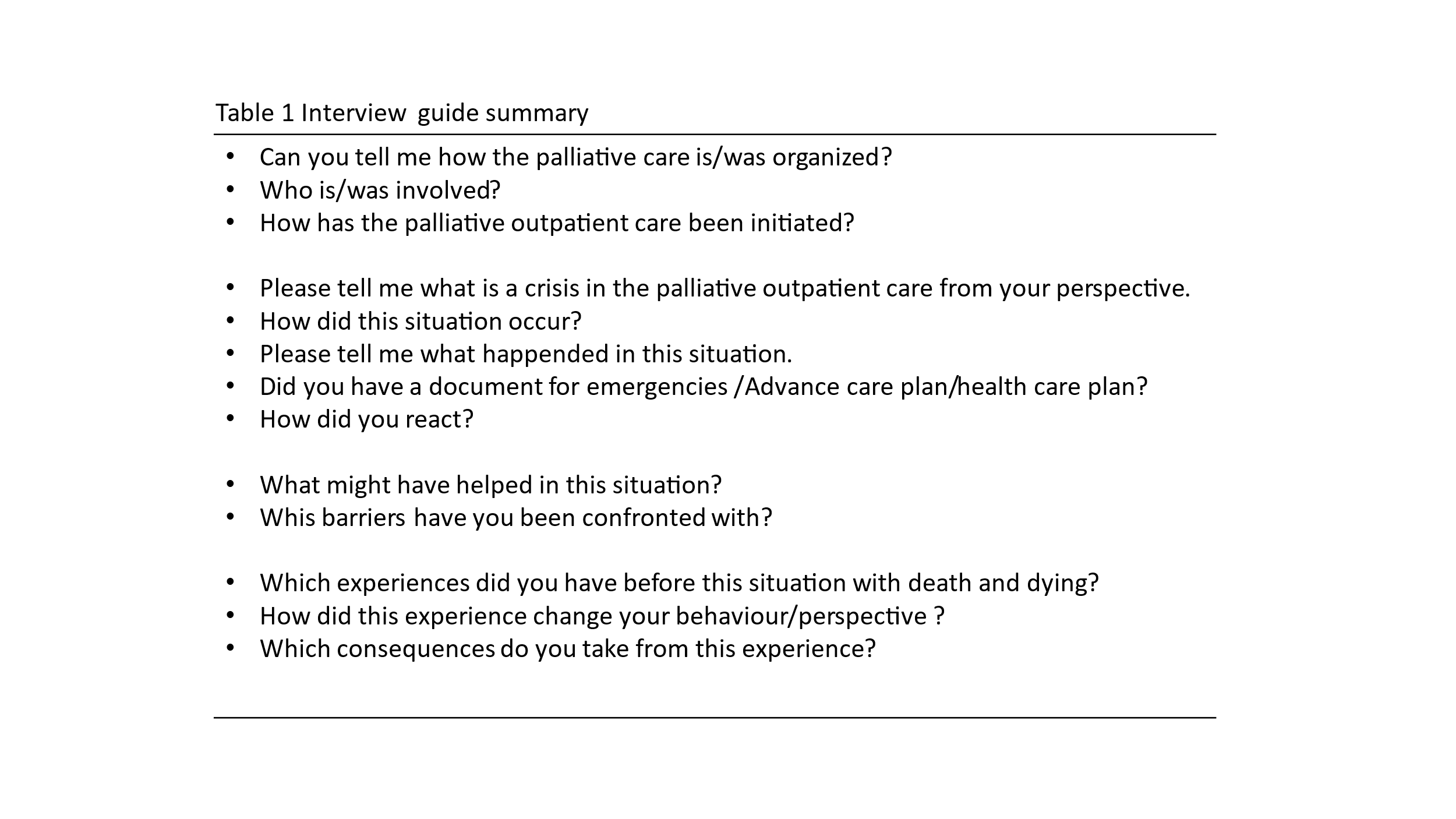
Table 1. Interview Guide English Version

Supplement: Supplementary file 1 — Additional file 1. Interview guide. [file 12904_2025_1664_MOESM1_ESM.docx]
